# Translational Control across Eukaryotes

**DOI:** 10.1155/2012/317690

**Published:** 2012-07-12

**Authors:** Greco Hernández

**Affiliations:** Division of Basic Research, National Institute for Cancer (INCan), Avenida San Fernando No. 22, Tlalpan, C.P. 14080, Mexico City, Mexico

Diversity is one of the most significant features of life. The current assessment of biodiversity on Earth reaches several millions of living species, and an unimaginable number of species that have gone extinct. Further more, diversity is ingrained in all aspects of life, namely, genetics, metabolic needs, cell complexity, body plans and organismal morphology, developmental programs, behavioral patterns, and the occupancy of ecological niches. A deep understanding of all levels of organismal diversity is necessary to obtain a profound understanding of life. For this reason, the main goal of this special issue on translational control across eukaryotes is to acknowledge and discuss recent research showing that molecular and functional diversification also exists in the translation apparatus of eukaryotes. “Translation,” that is, the process of decoding a messenger RNA to synthesize a protein by the ribosome, is a fundamental process for all forms of life.

The basic components and the global process of translation in eukaryotes have been largely established upon the study of traditional, so-called model organisms. However, this long-established overemphasis on model organisms has limited our knowledge of this process to few species. In recent years, the use of modern whole-genome sequencing and high-throughput technologies to study many nonmodel eukaryotes from disparate taxa has shown that the gene diversity in nature is far more expansive than we have ever imagined. These studies have uncovered a surprising diversity in the configuration of the translation apparatus across eukaryotes. This special issue acknowledges the diversity of translation factors in different lineages, the diversity found in key elements of mRNAs and RNA-binding proteins, and the diversity found in key RNA metabolism processes coupled to translational control, such as the RNA transport and the storage and degradation of mRNAs in cytoplasmic bodies. The first tutorial article, written by the editors of this installment, introduces the global topic as well as the spirit of the issue. Firstly, it introduces the general process of translation in eukaryotes and, secondly, it reviews the current knowledge on the many components of the translation machinery that have undergone diversification across phyla. 

Diversification of initiation factors is perhaps the most remarkable feature of the evolution of the translation apparatus. The papers by R. Jagus's group (University of Maryland, Baltimore, USA) and by A. Zinoviev and M. Shapira (Ben-Gurion University of the Negev, Beer Sheva, Israel) review this topic in protists, the most diverse but less studied group of eukaryotes. The paper by R. M. Patrick and K. S. Browning (University of Texas at Austin, USA) covers the role of the key initiation factors eIF4F and eIFiso4F in plants. It also traces the evolutionary history of these two complexes across the kingdom of plants. A. Muñoz and M. M. Castellano (Polytechnic University of Madrid, Spain) analyze the different mechanisms regulating translation initiation in plants in response to abiotic stresses. They describe the mechanisms that plants share with other eukaryotes as well as the plant-specific ones. G. Hernández (National Institute for Cancer, Mexico City), in collaboration with P. Lasko and N. Sonenberg (McGill University, Montreal, Canada) investigate the evolutionary pattern that eIF4E has undergone in the class Insecta. Insects comprise the majority of extant animal species described and are the most diverse animal group on the planet. They have a huge impact on the biosphere as well as in all aspects of human life and economy. 

In the last years, the role of the 5′-untranslated region of mRNA in translation regulation has been extensively studied. E. Martínez-Salas et al. (Autonomous University of Madrid, Spain) goes through internal ribosome entry sites (IRESs) as major elements regulating translation, specially under stress conditions, in many phyla. L. O. F. Penalva's group (University of Texas, San Antonio, USA) explores the different, non-IRES, regulatory elements present at this region of the mRNA.

L. Wurth (Centre for Genomic Regulation, Barcelona, Spain) reviews the role and the conservation in different eukaryotes of key RNA-binding proteins (RBPs), which regulate translation to adjust cell proteome to different environmental conditions. As discussed by Wurth and by G. Grech and M. Lindern (University of Malta, Malta), some RBP are emerging as major players in the development of different tumors. C. Gamberi and P. Lasko (McGill University, Montreal, Canada) discuss the importance and evolutionary conservation of another RBP that plays crucial roles in development as well as in other cellular processes, that is, the Bic-C family of proteins and some of their protein partners.

Translational control in eukaryotes is tightly coupled to several components and processes of cell metabolism. One of them is the RNA transport, which establishes cellular asymmetries of protein synthesis to ensure different cell process or developmental programs to occur. The RNA transport machineries have also diverged in different phyla and, together with them, some components of the translation apparatus also diverged. This topic is reviewed by P. Vazquez-Pianzola and B. Suter (University of Bern, Switzerland), who analyze the conservation and divergence across eukaryotes of two major RNA transport machineries in unicellular and multicellular eukaryotes, namely, the yeast “Locasome” and the *Drosphila* Bic-D/Egl/Dyn complex, respectively. Another fundamental aspect of the translational control coupled to RNA metabolism is the storage and degradation of mRNAs in different cytoplasmic bodies, such as processing bodies and stress granules, which are collections of ribonucleoprotein complexes containing translation factors. Current research on the diversity of these foci in different phyla is analyzed by C. Layana, P. Ferreo and R. Rivera-Pomar from the National University of La Plata (Argentina).

The studies and reviews presented in this issue complement research published in other journals. Collectively, these studies show that after eukaryotes emerged, both components and regulatory mechanisms of the translation apparatus continued evolving during eukaryotes diversification. This supports the idea that this apparatus is far from being evolutionarily static.

*Greco Hernández* *Greco Hernández*

